# Relationship of Vitamin D, Spondyloarthritis Activity and Left Ventricular Systolic and Diastolic Function

**DOI:** 10.3390/jcm15156017

**Published:** 2026-08-02

**Authors:** Silvija Miletić Gršković, Iva Uravić Bursać, Vedrana Tudor Špalj, Vedrana Drvar, Vlatka Sotošek, Tatjana Kehler, Viktor Peršić, Tamara Sinožić, Gordana Laškarin

**Affiliations:** 1 Hospital for Medical Rehabilitation of Hearth and Lung Diseases and Rheumatis “Thalassotherapia-Opatija”, M. Tita 180, 51410 Opatija, Croatia; 2 Department of Family Medicine, Faculty of Medicine, University of Rijeka, Brace Branchetta 20, 51000 Rijeka, Croatia; 3 Clinical Department of Laboratory Diagnostics, Clinical Hospital Center Rijeka, Vjekoslava Dukića 7, 51000 Rijeka, Croatia; 4 Department of Anesthesiology, Reanimatology, Emergency and Intensive Care Medicine, Medical Faculty, University of Rijeka, Braće Branchetta 20, 51000 Rijeka, Croatia; 5 Department of Clinical Medical Sciences II, Faculty of Health Studies, University of Rijeka, Viktora Cara Emina 2, 51000 Rijeka, Croatia; 6 Department of Medical Rehabilitation, Faculty of Medicine, University of Rijeka, B. Branchetta 20, 51000 Rijeka, Croatia; 7 Department of Physiology, Immunology and Pathophysiology, Faculty of Medicine, University of Rijeka, B. Branchetta 20, 51000 Rijeka, Croatia

**Keywords:** Ankylosing Spondylitis Disease Activity Score, Bath Ankylosing Spondylitis Functional Indeks, Disease Activity Index for Psoriatic Arthritis, echocardiography, spondyloarthritis, vitamin D

## Abstract

**Background/Objectives:** Vitamin D insufficiency is common in patients with spondyloarthritis (SpA). We analyzed the relationship between vitamin D, disease activity, and left ventricular (LV) function in patients with SpA. **Methods:** A total of 298 patients with SpA and 57 healthy controls were enrolled. Serum vitamin D and laboratory cardiovascular risk factors were analyzed, and LV function was assessed by echocardiography. Vitamin D, the Ankylosing Spondylitis Disease Activity Score (ASDAS), and the Disease Activity Index for Psoriatic Arthritis (DAPSA) were examined for mutual correlations using univariate and multivariate linear regression models and were compared between groups. **Results:** Vitamin D levels did not differ between the SpA group and controls, although vitamin D supplementation was more frequent among SpA patients. Vitamin D was not associated with SpA activity. ASDAS and DAPSA were positively correlated with isovolumic relaxation time, and DAPSA was inversely correlated with peak early diastolic mitral annular velocity (e′), after adjustment for age, sex, metabolic factors, and/or therapy. Patients with insufficient serum vitamin D levels (<75 nmol/L) showed greater impairment of systolic and diastolic LV function and a diabetic-like lipid profile compared with those with sufficient vitamin D levels. The e′ wave was positively correlated with vitamin D after controlling for confounding factors. These findings in patients with SpA are consistent with those previously reported in patients with psoriatic arthritis (PsA). **Conclusions:** Patients with SpA were characterized by vitamin D supplementation, increased disease activity parameters, and impaired LV function. SpA activity, independent of vitamin D, was associated with LV functional impairment, and vitamin D was associated with an increase in the e′ wave after adjustment for confounding factors. However, causal inferences cannot be drawn from correlational analyses.

## 1. Introduction

Spondyloarthritis (SpA) is a chronic, progressive inflammatory rheumatic disease [1]. Pain and stiffness due to enthesitis and arthritis in the spine and pelvic joints characterize the axial form of the disease (axSpA), including nonradiographic axSpA (nr-axSpA) and radiographic axSpA (r-axSpA) [2]. Peripheral spondyloarthritis (pSpA) typically affects larger joints in an asymmetrical pattern (oligoarthritis) and is frequently associated with enthesitis, particularly of the lower extremities, in HLA-B27-positive patients with reactive arthritis and in B27+ carriers [3]. In addition, pSpA commonly involves the distal interphalangeal joints of the hands and feet and may be accompanied by dactylitis, particularly in HLA-Cw6-positive patients [3]. Both pSpA and axSpA may occur in patients without features of other SpA subtypes (pure pSpA or undifferentiated axSpA, respectively) and impose a significant burden on patients [4]. Enteropathic peripheral arthritis is more frequently associated with intestinal inflammation, although all forms of axSpA may occur in the presence of clinically recognized or unrecognized gastrointestinal tract inflammation [5]. The gastrointestinal tract is a cradle of SpA inflammation [6], which is inversely correlated with circulating 25-hydroxycholecalciferol (25OHD3) concentration [7].

Vitamin D3, or cholecalciferol, is a form of vitamin D produced in human skin from cholesterol under the influence of UV radiation. In the liver, it is hydroxylated to 25OHD3, which then enters and circulates in the blood [8]. Owing to its relatively long half-life of approximately 2–3 weeks, serum 25OHD3 is routinely measured to assess vitamin D status [9].

Under physiological conditions, renal epithelial cells further hydroxylate 25OHD3 at the 1α position, producing the biologically active hormone 1α,25-dihydroxycholecalciferol (1α,25OH2D3) [10], which plays a central role in calcium and phosphate homeostasis. Concentrations of 1α,25OH2D3 are markedly reduced in patients with chronic kidney disease and α-Klotho deficiency, an early marker of renal dysfunction involved in phosphate, calcium, and vitamin D metabolism [11].

Circulating 25OHD3 also enters antigen-presenting cells and T lymphocytes, where it is converted to 1α,25OH2D3 by 1α-hydroxylase. This active metabolite subsequently binds to the vitamin D receptor, a cytoplasmic and nuclear receptor whose expression increases following immune cell activation [12]. Through this mechanism, vitamin D exerts profound immunomodulatory effects by regulating T-cell differentiation [13]. Specifically, 1α,25OH2D3 suppresses interleukin-23 (IL-23) production by dendritic cells—a cytokine indispensable for Th17-cell polarization [7]—and activation of the IL-23/IL-17 axis, which plays a central role in the pathogenesis and exacerbation of SpA [6], including both musculoskeletal [14] and extra-musculoskeletal manifestations [15]. These findings suggest that 1α,25OH2D3 contributes to the downregulation of ongoing inflammatory responses in SpA [6,14,15].

Vitamin D deficiency is associated with increased activity of r-axSpA and PsA [16] and is negatively correlated with all-cause mortality [17], of which cardiovascular (CV) disease represents the major contributor [18]. A high incidence of endothelial dysfunction and traditional CV risk factors such as obesity and metabolic syndrome are associated with high PsA activity [19]. Increased left ventricular (LV) mass, pressure and volume overload, and diastolic dysfunction have frequently been reported in patients with SpA [20]. Even in the absence of traditional risk factors, patients with PsA have a higher CV risk of myocardial infarction, stroke, and heart failure [21], and patients with r-axSpA exhibit LV systolic and diastolic dysfunction [22] as a result of disease activity and systemic inflammation [23]. 25OHD3 deficiency may be a marker of inflammatory activity in SpA [16], although it is not causally related to r-axSpA activity [24], and contradictory data exist regarding the relationship between vitamin D and SpA activity [25]. Limited and contradictory data also exist in the literature regarding the effects of 25OHD3 on LV function in patients with SpA.

The aim of this study was to analyze the relationships between vitamin D concentration, disease activity, and patient functionality, as well as to evaluate correlations between LV diastolic and systolic function measured by echocardiography and serum 25OHD3 concentration (hereinafter referred to as vitamin D), the Ankylosing Spondylitis Disease Activity Score (ASDAS), an axSpA activity measure [26], and the Disease Activity Index for Psoriatic Arthritis (DAPSA), a pSpA activity index [27], in patients with SpA.

## 2. Materials and Methods

### 2.1. Patients

This cross-sectional study was conducted at the Hospital for Medical Rehabilitation of Heart and Lung Diseases and Rheumatism, “Thalassotherapia Opatija,” in Croatia, between February 2021 and November 2025. The study included 298 patients with all SpA subtypes, diagnosed according to the guidelines/criteria of the International Society for the Evaluation of Spondyloarthritis [28], who were regularly followed at an outpatient rheumatology clinic. A rheumatologist assessed disease activity and functional status for each patient. The control group consisted of age- and sex-matched individuals without SpA, recruited during routine medical examinations at Thalassotherapia Opatija. Both patients and controls underwent laboratory testing and a comprehensive cardiological evaluation.

Prior to participation, all subjects provided written informed consent, confirming their voluntary participation in the study and authorizing the research team to access relevant medical records and publish anonymized data. The informed consent forms for both SpA patients and controls were approved by the Ethics Committee of Thalassotherapia Opatija (Approval No. 01-000-00-309/2-2023; 20 April 2023).

The study was conducted in accordance with the principles of the Declaration of Helsinki of the World Medical Association [29] and all applicable regulations designed to ensure participant safety and confidentiality.

Exclusion criteria for both groups were infection; malignant disease; unregulated diabetes (fasting glucose above 11 mmol/L); ambulatory arterial blood pressure (BP) > 160/100 mmHg, corresponding to home arterial BP > 145/90 mmHg [30]; heart failure with New York Heart Association class III or IV [31]; and chronic kidney disease with an estimated glomerular filtration rate (eGFR) below 15 mL/min/1.73 m^2^. Patients did not change their regular cardiological and/or rheumatological therapy during the course of the study.

### 2.2. Rheumatological Examination

Pondyloarthritis (SpA) activity was evaluated using patient-reported outcomes and findings from rheumatological examination. Patient-reported disease burden was assessed by measuring total pain and axial pain using a visual analog scale (VAS; 0–100 mm) and a numerical rating scale (NRS; 0–10), respectively. A comprehensive rheumatological examination was performed, during which the components required to calculate the Disease Activity in Psoriatic Arthritis index (DAPSA), the Ankylosing Spondylitis Disease Activity Score (ASDAS), and the Bath Ankylosing Spondylitis Disease Activity Index (BASDAI) were assessed. Functional status was evaluated using the Bath Ankylosing Spondylitis Functional Index (BASFI).

These clinical parameters, together with the laboratory marker C-reactive protein (CRP), were used to calculate composite indices of disease activity in both the axial and peripheral forms of SpA: ASDAS for axial disease [26] and DAPSA for peripheral disease [27]. The ASDAS incorporates back pain severity, peripheral pain and joint swelling, duration of morning stiffness, and the patient’s global assessment of SpA activity, all measured on a VAS ranging from 0 to 10. The DAPSA score comprises tender joint count (0–68), swollen joint count (0–66), patient global assessment of disease activity, and overall joint pain during the preceding week (VAS 0–10).

Additionally, SpA activity was assessed using the composite scales BASDAI and BASFI [28]. BASDAI evaluates the overall severity of fatigue; neck, back, and hip pain attributable to axial SpA; pain and swelling in peripheral joints; discomfort in areas tender to touch or pressure; and symptom severity upon awakening, all measured on a VAS ranging from 0 to 10. The duration of morning stiffness is also included in the assessment. BASFI consists of ten questions evaluating the degree of difficulty in performing daily activities without assistance or assistive devices. These activities include putting on socks or tights, bending from the waist to pick up an object from the floor, reaching a high shelf, rising from an armless chair, getting up from the floor from a supine position, standing for 10 min without discomfort, climbing 12–15 steps, looking over the shoulder without turning the body, performing physically demanding activities (e.g., exercise or gardening), and carrying out a full day of activities at home or work. Each item is scored on a VAS ranging from 0 to 10 [32].

Demographic data (age and sex), medical history, and current therapy were obtained from the hospital information system (WinBis, IN2 d.o.o., Zagreb, Croatia).

### 2.3. Cardiological Examination and Echocardiography

A cardiologist recorded age, sex, and current therapy, measured blood pressure, calculated body mass index (BMI), and performed echocardiography in patients with SpA and in healthy controls. Transthoracic echocardiography was performed in accordance with the recommendations of the American Society of Echocardiography and the European Association of Cardiovascular Imaging [33,34], using a GE Vivid 9 system (General Electric Company, New York, NY, USA) and a GE M5Sc-D phased-array probe (1.4–1.6 MHz; Parallel Design SAS, Valbonne, France). Parameters of LV systolic function—including left ventricular ejection fraction (LVEF), global longitudinal strain (GLS), and mitral annular plane systolic excursion (MAPSE)—as well as the structural parameter LV end-diastolic diameter (LVEDD), were assessed. LVEDD was measured in the apical four-chamber view at end-diastole. MAPSE was determined using M-mode echocardiography at the septal mitral annulus in the apical four-chamber view. LVEF was calculated using the biplane Simpson method, while GLS was assessed using two-dimensional (2D) speckle-tracking echocardiography. Peak tricuspid regurgitation velocity (TR velocity) was measured from the apical four-chamber view. Left atrial volume indexed to body surface area (LAVI) was calculated using the biplane method of disks.

Diastolic function was evaluated by pulsed-wave Doppler assessment of mitral inflow velocities, obtained in the apical four-chamber view at the tips of the mitral valve leaflets. Measured parameters included peak early diastolic mitral inflow velocity (E-wave), peak late diastolic mitral inflow velocity (A-wave), isovolumic relaxation time (IVRT), and the E/A ratio. Tissue Doppler imaging (TDI) was used to assess peak early diastolic mitral annular velocity (e′) and the E/e′ ratio. Left atrial deformation parameters included peak left atrial reservoir strain (LASr), peak strain rate during the conduit phase (SrE), and peak strain rate during the contractile phase (SrA); these were measured from the apical four-chamber view using 2D speckle-tracking echocardiography. Strain analysis was performed using EchoPAC software (GE Healthcare, Chicago, IL, USA). The left atrial endocardial border was manually traced at end-systole in the apical four-chamber view. LASr was defined as the maximal positive peak on the left atrial strain curve generated by the software, while SrE and SrA were defined as the peak negative strain rates during the conduit and contractile phases, respectively.

LV GLS analysis was performed using apical two-, three-, and four-chamber views, with the final GLS value calculated as the average across all segments. All echocardiographic data were digitally stored and analyzed offline using EchoPAC software (GE Healthcare, Chicago, IL, USA).

### 2.4. Laboratory Analysis

A peripheral venous blood sample (10 mL) was obtained from the cubital vein by venipuncture for laboratory analyses. Erythrocyte sedimentation rate (ESR), C-reactive protein (CRP), vitamin D, urea, uric acid, creatinine, total cholesterol, high-density lipoprotein cholesterol (HDL-C), fasting blood glucose (FBG), N-terminal pro-brain natriuretic peptide (NT-proBNP), and cardiac troponin T concentrations were measured using an automated analyzer (Cobas^®^ Pro, Roche Diagnostics, Mannheim, Germany). Estimated glomerular filtration rate (eGFR) was calculated as a measure of renal function.

Serum samples were separated, and a 200 μL aliquot was transferred into a cryotube (2 mL; Falcon, Corning Inc., NY, USA) and stored at −20 °C until analysis. Serum Klotho protein concentration was determined in undiluted serum samples using a commercially available Human KL (Klotho) ELISA kit (Cat. No. ELK3157; ELK Biotechnology, Sugar Land, TX, USA), according to the manufacturer’s instructions. Absorbance was measured using a microplate reader, and concentrations were calculated using CurveExpert software (version 1.40).

### 2.5. Statistical Analyses

Sample size estimation was performed using preliminary data from 15 participants and the online Sample Size Calculator developed by Ristl R. (version 1.064, University of Vienna; accessed 2 May 2023).

Pearson’s correlation coefficient (r) was used for univariate correlation analyses. Multiple linear regression analyses were performed to determine whether vitamin D independently predicted SpA activity parameters, and whether ASDAS and DAPSA independently predicted LV function parameters. The regression models were adjusted for potential confounders, including sex, age, total cholesterol, low-density lipoprotein cholesterol (LDL-C), high-density lipoprotein cholesterol (HDL-C), fasting blood glucose (FBG), uric acid, creatinine, estimated glomerular filtration rate (eGFR), systolic and diastolic blood pressure, and cigarette smoking status. Multivariate linear regression analyses were performed using several models, each comprising a different combination of confounders, as the small sample sizes within individual correlation groups did not allow all confounders to be analyzed in a single model. Statistical analyses were conducted using IBM SPSS Statistics, version 22 (IBM Corp., Armonk, NY, USA). Correlation coefficients with an absolute value below 0.25 were considered to indicate a weak or negligible linear relationship [35].

All other statistical analyses were performed using Statistica, version 14.0.0.15 (TIBCO Software Inc., Palo Alto, CA, USA). Continuous variables were tested for normality using the “Distribution Fitting” and “Continuous Distribution” options and are presented as mean ± standard deviation (SD) for normally distributed data or median (range) for non-normally distributed data. Between-group comparisons were performed using the independent-samples Student’s *t*-test for normally distributed variables and the Mann–Whitney U test for non-normally distributed variables. The Mann–Whitney U test was also applied when group sample sizes were small (n < 50).

Categorical variables are presented as frequencies and percentages. Comparisons between categorical variables were performed using Fisher’s exact test. A two-sided *p*-value < 0.05 was considered statistically significant for all analyses.

## 3. Results

### 3.1. Recruitment, Clinical Protocol and Characteristics of the Assessment Groups

Based on the observed correlation between vitamin D concentration and DAPSA score (r = 0.26), with a significance level (α) of 0.05 and a statistical power of 80%, the required sample size was estimated to be 114 participants. For the correlation between vitamin D and LVEF (r = 0.457), the required sample size was 36; for GLS (r = 0.611), 19; for LASr (r = 0.273), 204; for the e′ wave (r = 0.526), 26; for IVRT (r = 0.424), 42; and for MAPSE (r = 0.597), 20.

**Figure 1:**
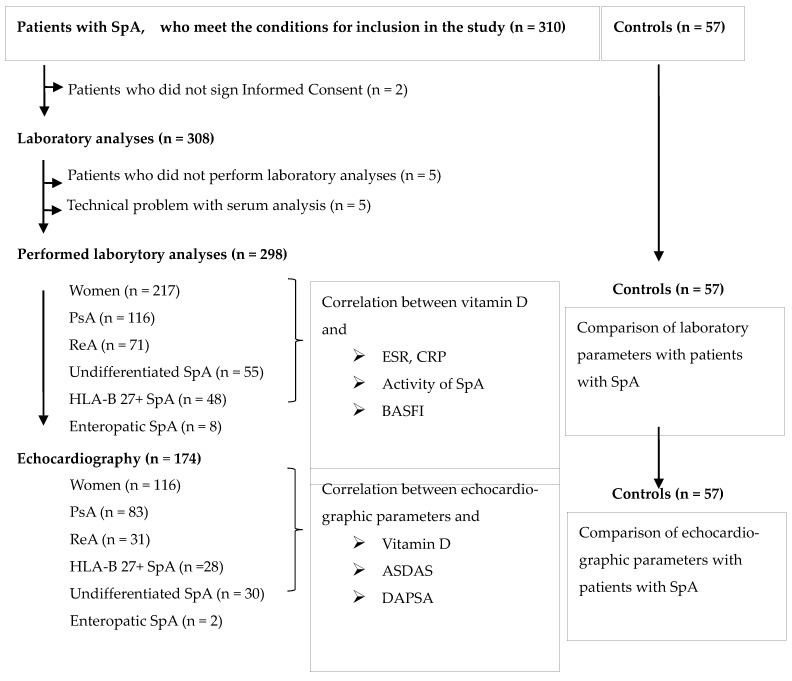
Recruitment, clinical protocol and characteristics of the assessment groups. Abbreviations: ASDAS—Axial Spondyloarthritis Disease Activity Score; BASFI—Bath Ankylosing Spondylitis Functional Index; CRP—C-reactive protein; DAPSA—Disease Activity index for Psoriatic Arthritis; ESR—Erythrocyte Sedimentation Rate; HLA—B27+—Human Leukocyte Antigen B 27 positive; n—number; %—percentage; PsA—Psoriatic Arthritis; ReA—Reactive Arthritis; SpA—Spondyloarthritis.

Figure 1 shows the recruitment of patients with SpA (n = 310) and healthy controls (n = 57) into the study groups. Two patients with SpA did not sign informed consent, leaving 308 patients eligible for laboratory blood analysis. Five patients did not undergo laboratory testing, and serum samples from an additional five patients were not properly analyzed for technical reasons. These patients were excluded, resulting in a final study population of 298 patients with SpA.

Women accounted for 73% (n = 217/298) of the study population. Axial SpA (axSpA) was present in 64% of patients, comprising 46% radiographic axSpA (r-axSpA) and 52% non-radiographic axSpA (nr-axSpA), while peripheral SpA (pSpA) accounted for 36% of cases. The study cohort included patients with psoriatic arthritis (PsA; n = 116, 39%), reactive arthritis (ReA; n = 71, 24%), undifferentiated SpA (n = 55, 18%), HLA-B27-positive SpA (n = 48, 16%), and enteropathic arthritis (n = 8, 3%). In this cohort, the relationships between vitamin D concentration and ESR, CRP, disease activity indices, and functional status (BASFI) were evaluated.

A randomly selected subgroup of 174 patients with SpA who had completed laboratory testing underwent echocardiographic assessment. Women represented 67% of this subgroup (n = 116/174). The subgroup consisted of patients with PsA (n = 83, 48%), ReA (n = 31, 18%), undifferentiated SpA (n = 30, 17%), HLA-B27-positive SpA (n = 28, 16%), and enteropathic arthritis (n = 2, 1%). The distribution of SpA subtypes, sex, and age (58.1 ± 11.3 years) did not differ significantly from that of the overall SpA cohort (56.0 ± 11.9 years) (Table 1). In this subgroup, associations between vitamin D concentration, ASDAS, DAPSA, and echocardiographic parameters were analyzed. Furthermore, laboratory, clinical, and echocardiographic characteristics were compared between patients with SpA and the control group.

### 3.2. Clinical, Laboratory and Echocardiographic Characteristics of Patients with SpA

Table 1 shows the clinical, laboratory, and echocardiographic findings in patients with SpA and controls. Clinical parameters such as age and BMI, and laboratory parameters such as ESR, CRP, and total cholesterol, did not differ significantly between patients with SpA and controls. HDL-C level was lower and FBG was higher in the SpA group (*p* = 0.0003 and *p* = 0.0014, respectively). Uric acid, eGFR, and vitamin D did not differ significantly. Systolic and diastolic BP were higher in patients with SpA than in controls (*p* = 0.0060 and *p* = 0.0155, respectively). No significant difference was observed in SCORE2/SCORE2-OP values between the groups.

The echocardiographic parameters LVEF and MAPSE were lower (*p* = 0.0083 and *p* = 0.0024, respectively), and GLS was worse (less negative) (*p* = 0.0037), in patients with SpA than in controls. LVEDD and the A-wave did not differ significantly. The E-wave and E/A ratio were decreased in patients with SpA compared with controls (*p* < 0.0001 and *p* = 0.0037, respectively). LAVI did not differ significantly. LASr (*p* < 0.0001) and the e′-wave (*p* = 0.0364) were lower in patients with SpA than in controls. The E/e′ ratio did not differ. SrE was lower in patients with SpA (*p* < 0.0001), whereas SrA did not differ significantly. IVRT was higher in patients with SpA (*p* < 0.0001). No significant differences were observed in troponin T, NT-proBNP, or Klotho concentrations between the groups.

### 3.3. Comorbidities and Current Therapy of Patients with SpA

Table 2 shows the comorbidities and current therapy of patients with SpA and controls. Arterial hypertension (*p* < 0.0001), hyperglycemia (*p* = 0.0483), osteoarthritis (*p* < 0.0001), cigarette smoking (*p* = 0.0008), and vitamin D supplementation (*p* < 0.0001) were more prevalent in patients with SpA than in controls. The frequency of hyperlipidemia did not differ significantly between the groups, while statin use was more frequent in patients with SpA (*p* = 0.0156). The use of nonsteroidal anti-inflammatory drugs (NSAIDs; *p* < 0.0001), conventional synthetic disease-modifying antirheumatic drugs (csDMARDs; *p* < 0.0001), biologic disease-modifying antirheumatic drugs (bDMARDs; *p* = 0.0155), beta-blockers (*p* = 0.0057), and angiotensin-converting enzyme inhibitors/angiotensin II receptor blockers (ACE inhibitors/ARBs; *p* = 0.0452) were all more common among patients with SpA than in the control group. Treatment with calcium channel blockers, diuretics, and antidiabetic medications did not differ significantly between the groups.

### 3.4. Differences in Patients with SpA with Respect to the Vitamin D Concentration

Table 3 presents a comparison of echocardiographic and metabolic parameters between patients with SpA, stratified according to serum vitamin D concentration using the lower limit of the physiological range (75 nmol/L) as the cutoff value. The mean ± SD serum vitamin D concentration in the vitamin D-insufficient group (n = 183) was 53.0 ± 13.5 nmol/L. In the group with physiological vitamin D concentrations (n = 77), the mean concentration was 90.2 ± 14.8 nmol/L.

HDL-C was lower (*p* = 0.0009) and triglyceride level was higher (*p* = 0.01) in patients with vitamin D insufficiency compared with patients with physiological serum vitamin D concentrations. LDL-C, total cholesterol, FBG, uric acid, and creatinine concentrations did not differ significantly between the groups. The systolic function parameters LVEF and LVEDD did not differ significantly between the groups. MAPSE was lower and GLS was impaired (i.e., less negative) in patients with vitamin D insufficiency versus patients with serum vitamin D > 75 nmol/L (*p* = 0.0166 and *p* = 0.0421, respectively). The diastolic function parameters E-wave, A-wave, E/A ratio, TR velocity, LAVI, SrE, SrA, and E/e′ ratio did not differ significantly between patients grouped according to serum vitamin D concentration. The e′-wave, LASr, and IVRT were all lower in patients with vitamin D insufficiency than in patients with physiological serum vitamin D concentrations (*p* = 0.0044, *p* = 0.0050, and *p* = 0.0052, respectively).

### 3.5. Correlations of Vitamin D and Indices of Spondylarthritis Activity with Echocardiographic Parameters of LV Function in Patients with Spondylarthritis

**Figure 2:**
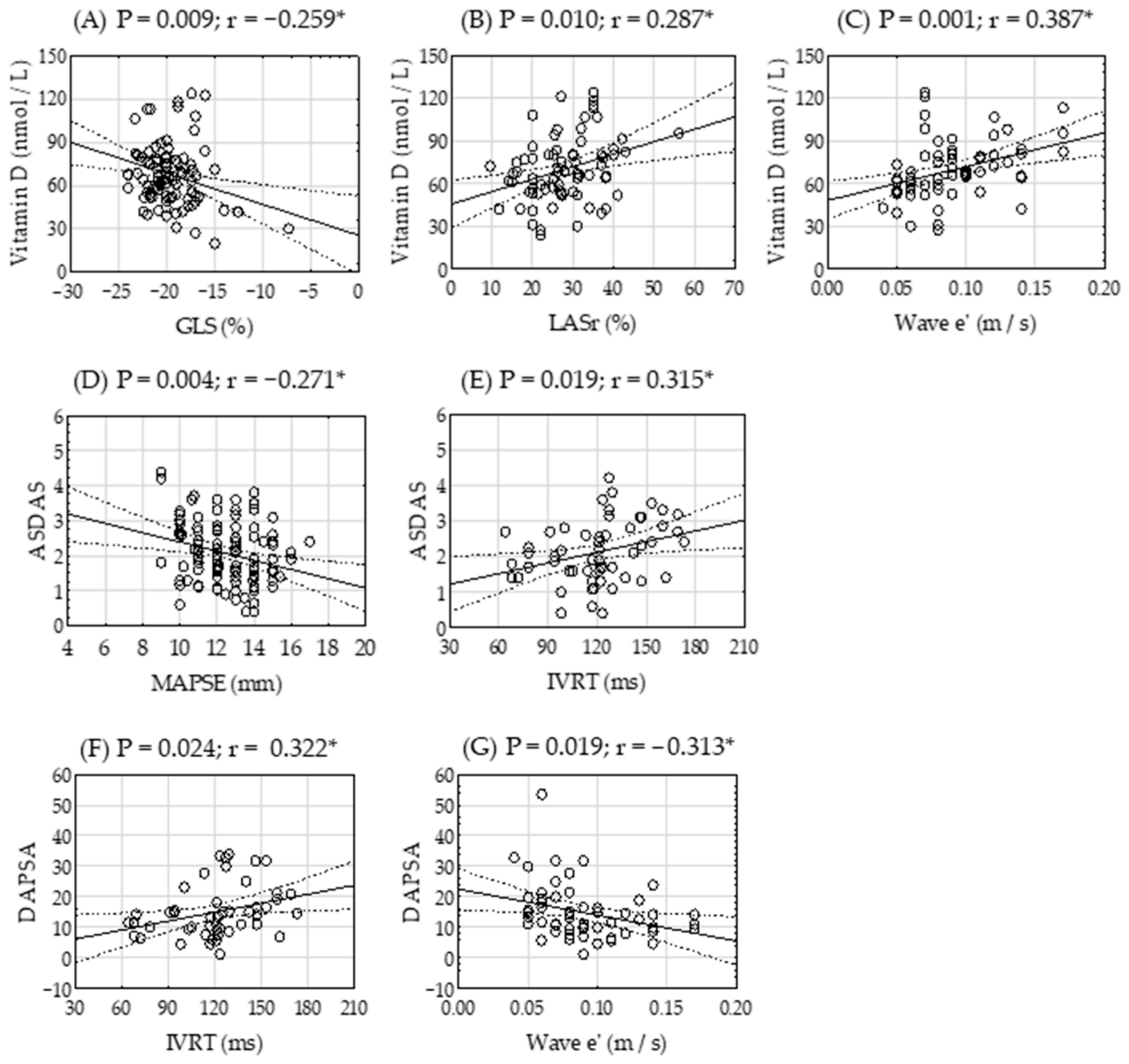
Correlation of echocardiographic parameters, vitamin D, Axial Spondyloarthritis Disease Activity Score [ASDAS] and Disease Activity Index for Psoriatic Arthritis [DAPSA] in patients with spondyloarthritis [SpA], n 298. The correlations of vitamin D and (**A**) global longitudinal strain [GLS]; (**B**) peak systolic left atrial reservoir strain [LASr] and (**C**) early diastolic mitral annular velocity [wave e′] are shown in dot plots of the upper line. The correlations of ASDAS and (**D**) mitral annular plane systolic excursion [MAPSE] and (**E**) isovolumic relaxation time [IVRT] are shown in the dot plots of the middle line. The correlation of DAPSA and (**F**) IVRT and (**G**) wave e′ are shown in the dot plots of the lower line. Level of significance (*p*) less than 0.05 and correlation coefficients [r] > 0.25 were considered significant in Pearson univariate analysis. Significance is shown with asterisks [*] above scatter plots. The number of paired parameters for correlation was from 49 to 110.

Figure 2 shows the relationships among vitamin D, ASDAS, and DAPSA and selected parameters of LV function in patients with SpA.

In univariate analysis, vitamin D concentration showed a weak negative correlation with GLS (*p* = 0.009, r = −0.259) (Figure 2A). This correlation: (I) remained significant (r = −0.327, *p* = 0.006) and independent of the influence of metabolic factors (FBG, LDL-C, HDL-C), systolic BP, or statin therapy in multivariate linear regression model I; (II) remained significant (r = −0.390, *p* = 0.001) and independent of FBG, LDL-C, systolic BP, or NSAID therapy in multivariate linear regression model II; and (III) became nonsignificant (*p* = 0.146, β = −0.172) after adjustment for sex, age, uric acid, creatinine, fasting blood glucose, and smoking status in multivariate linear regression model III. Age (*p* = 0.049) and creatinine concentration (*p* = 0.028) emerged as significant predictors of GLS. The overall model explained 21% of the variance in GLS (*p* = 0.044), with age and creatinine accounting for approximately 6% and 7% of the variance, respectively.

A weak positive correlation was found between vitamin D concentration and LASr in univariate analysis (*p* = 0.010, r = 0.287; Figure 2B), which remained significant independent of age and sex in multivariate linear regression model I (n = 78). However, in multivariate model II, which accounted for vitamin D, LASr, FBG, LDL-C, HDL-C, and statin therapy, the association between vitamin D and LASr disappeared. The association between vitamin D and HDL-C was significant (r = 0.315, *p* = 0.024) and independent of LASr, FBG, LDL-C, and statin therapy. The model including NSAID therapy was not statistically significant.

Vitamin D concentration also demonstrated a weak positive correlation with the e′ wave (*p* = 0.001, r = 0.387; Figure 2C), which remained significant independent of age and sex in the adjusted model (n = 69). The association between vitamin D and e′ remained significant (r = 0.438, *p* = 0.001) and independent of lipid parameters (LDL-C and HDL-C) or NSAID therapy in multivariate analysis. Multiple linear regression analysis demonstrated that the associations of vitamin D with the e′ wave and with LDL-C were significant (r = 0.416, *p* = 0.001 for the e′ wave; r = −0.237, *p* = 0.049 for LDL-C) and independent of HDL-C or statin therapy.

A significant but weak negative correlation between ASDAS and MAPSE was observed in univariate analysis (*p* = 0.004, r = −0.271; Figure 2D), which disappeared after adjustment for FBG and uric acid levels (model I) or after adjustment for FBG, systolic BP, LDL-C, HDL-C, and statin therapy (model II).

ASDAS showed a weak association with IVRT in univariate analysis (*p* = 0.019, r = −0.315; Figure 2E), which remained significant independent of age and sex in multivariate analysis (n = 55). The associations of ASDAS with IVRT and with HDL-C remained significant (r = 0.347, *p* = 0.013 for IVRT; r = −0.286, *p* = 0.038 for HDL-C) and independent of LDL-C or NSAID therapy. Similarly, the associations of ASDAS with IVRT and with HDL-C were significant (r = 0.316, *p* = 0.031 for IVRT; r = −0.286, *p* = 0.042 for HDL-C) and independent of LDL-C or statin therapy in multiple linear regression analysis.

A significant positive correlation was observed between DAPSA and IVRT (*p* = 0.024, r = 0.322; Figure 2F). The associations of DAPSA with IVRT and with LDL-C were also significant (r = 0.399, *p* = 0.009 for IVRT; r = 0.358, *p* = 0.018 for LDL-C) and independent of HDL-C or statin therapy. IVRT and LDL-C emerged as significant and independent predictors of DAPSA activity (r = 0.428, *p* = 0.005 for IVRT; r = 0.334, *p* = 0.027 for LDL-C), even after adjusting for HDL-C and NSAID therapy.

DAPSA was significantly negatively correlated with the e′ wave (*p* = 0.019, r = −0.313; Figure 2G). This association was not further influenced by age or sex (n = 56). The e′ wave emerged as a significant and independent predictor of DAPSA (r = −0.296, *p* = 0.039), even after adjusting for lipid parameters (LDL-C and HDL-C) and statin therapy. The model including NSAID therapy was not statistically significant.

Supplementary Table S1 shows correlations between vitamin D and inflammatory indicators (ESR, CRP, VAS for total pain, VAS for axial pain, ASDAS, DAPSA, BASDAI), the index of patient functionality (BASFI), and echocardiographic parameters such as LVEF, LVEDD, MAPSE, IVRT, and E/e′ ratio; none of these relationships was significant. Neither ASDAS nor DAPSA correlated with LVEF, GLS, LVEDD, LASr, E/e′ ratio, or the e′ wave (Supplementary Table S1). DAPSA also did not correlate with MAPSE (Supplementary Table S1).

### 3.6. Relationship of Vitamin D and Indexes of Spondyloarthritis Activity with Echocardiographic Parameters in Patients with Psoriatic Arthritis

**Figure 3:**
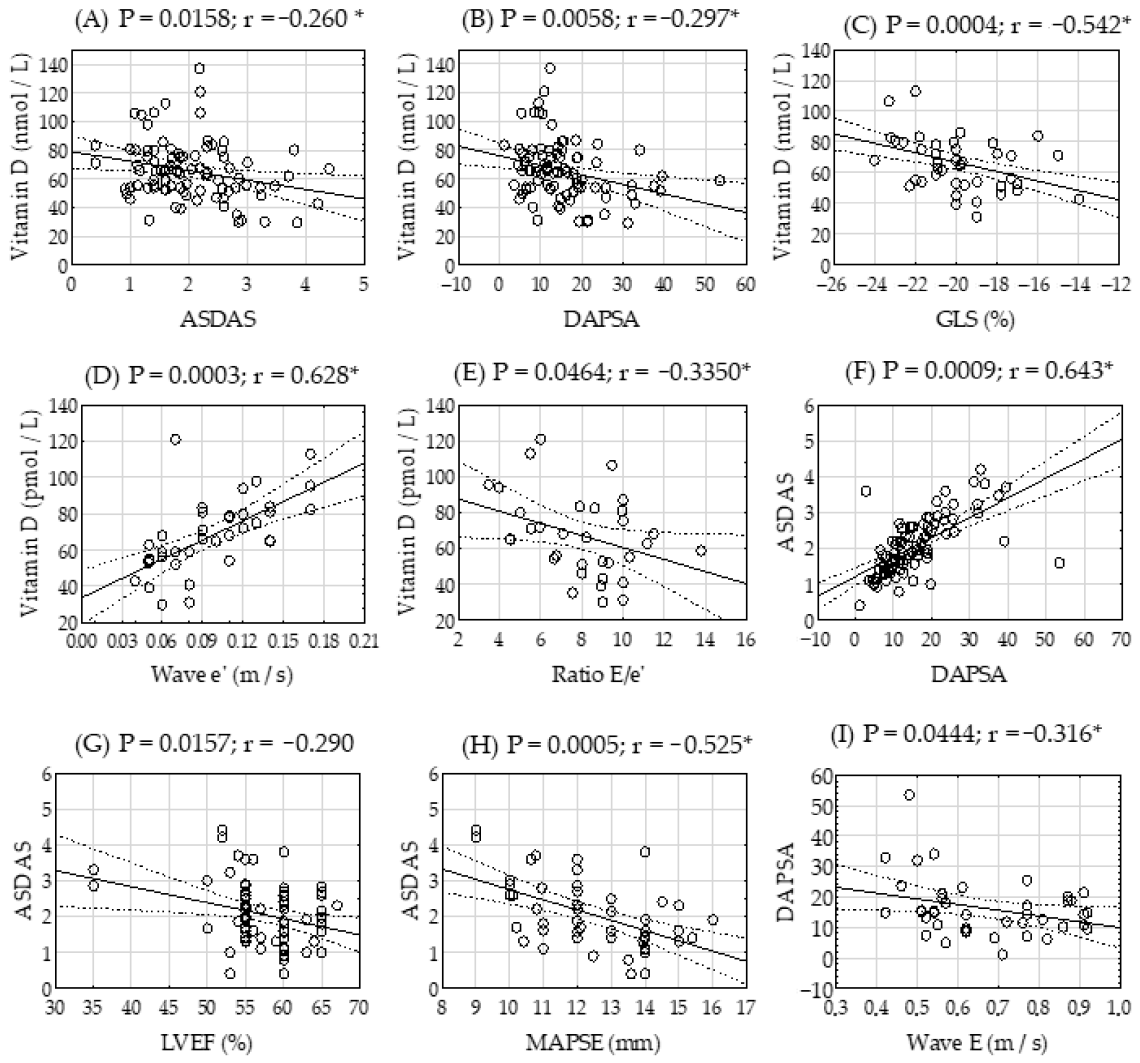
Correlation between left ventricular systolic and/or diastolic parameters, vitamin D, Axial Spondyloarthritis Disease Activity Score [ASDAS] and Disease Activity Index for Psoriatic Arthritis [DAPSA] in patients with Psoriatic Arthritis [PsA]. The correlations of vitamin D and (**A**) ASDAS; (**B**) DAPSA; (**C**) global longitudinal strain [GLS]; (**D**) early diastolic mitral annular velocity [wave e′]; (**E**) ratio E/e′; The correlations of ASDAS and (**F**) DAPSA; (**G**) left ventricular ejection fraction [LVEF] and (**H**) mitral annular plane systolic excursion [MAPSE]. Correlation of DAPSA and (**I**) peak early diastolic mitral inflow velocity [wave E]. Level of significance (*p*) less than 0.05 and correlation coefficients [r] > 0.25 were considered significant in Pearson univariate analysis. Significance is shown with asterisks [*] above scatter plots. The number of paired parameters for a particular correlation was from 36 to 88.

Figure 3 shows the interdependence of vitamin D and SpA activity parameters (ASDAS and DAPSA) with LV echocardiographic parameters in the subgroup of patients with SpA consisting of patients with PsA.

A statistically significant negative correlation was found between vitamin D and ASDAS (r = −0.260), DAPSA (r = −0.297), and GLS (r = −0.542) (Figure 3A, Figure 3B, and Figure 3C, respectively). In multivariate analysis, vitamin D was significantly correlated with GLS (*p* = 0.002) after controlling for FBG and statin and NSAID use, neither of which was significantly related (n = 40; R^2^ = 0.283; *p* = 0.018). However, the correlations between vitamin D and ASDAS, and between vitamin D and DAPSA, became nonsignificant after controlling for FBG, LDL-C, and statin or NSAID use.

Vitamin D was positively correlated with the e′ wave (*p* = 0.0003, r = 0.628; Figure 3D). Multivariate linear regression analysis confirmed that vitamin D was significantly correlated with the e′ wave (*p* < 0.001; 56% unique variance) and with statin use (*p* = 0.020; 8% unique variance) after controlling for FBG, which was not significantly correlated (N = 29; R^2^ = 0.668; *p* < 0.001). Vitamin D was inversely correlated with the E/e′ ratio (*p* = 0.0464, r = −0.335; Figure 3E). In multivariate analysis, this correlation was not significant after controlling for FBG and statin therapy.

ASDAS and DAPSA were significantly positively associated (r = 0.643; Figure 3F). ASDAS was correlated with DAPSA (43% unique variance; *p* < 0.001) after controlling for FBG, LDL-C, and statin or NSAID use, none of which significantly affected their relationship (N = 76; R^2^ = 0.507; *p* < 0.001). ASDAS was inversely associated with LVEF (*p* = 0.0157, r = −0.290; Figure 3G), and this correlation remained significant after controlling for FBG and statin use, neither of which significantly influenced it (N = 61; R^2^ = 0.149; *p* = 0.026). Additionally, ASDAS was negatively associated with MAPSE (*p* = 0.0005, r = −0.525; Figure 3H). In multivariate analysis, ASDAS was correlated with both MAPSE (10% unique variance; *p* = 0.016) and glucose (14% unique variance; *p* = 0.004) after controlling for statin use, which did not significantly affect the correlation (N = 44; R^2^ = 0.397; *p* < 0.001).

DAPSA was inversely correlated with the E-wave (*p* = 0.0444, r = −0.316; Figure 3I), although this correlation became nonsignificant after controlling for FBG and statin therapy.

Vitamin D, ASDAS, and DAPSA were not correlated with any other echocardiographic parameters of LV function (Supplementary Table S2).

## 4. Discussion

In patients with SpA, many circulating cytokines, including IL-17, IL-23, IL-1, IL-6, and tumor necrosis factor-alpha (TNF-α) [36,37,38,39], can incorporate into the endothelial glycocalyx and alter its physiological structure and function [40], thereby damaging the underlying endothelium. Damaged endothelium is the starting point for many diseases, including comorbidities of SpA such as hypertension, central obesity, diabetes, atherosclerosis, and vascular narrowing [41,42,43,44,45]. The more frequent use of NSAIDs, csDMARDs, and bDMARDs in patients with SpA than in controls is considered beneficial for the endothelium and may help prevent CV events through treatment of inflammation [43]. This is especially important in patients who smoke, as SpA severity is more pronounced in smokers [44], who were numerous among our patients with SpA. In this study, patients with SpA were more frequently diagnosed with arterial hypertension and hyperglycemia (diabetes or insulin resistance) than controls, consistent with an increased classic CV risk [23,46]. Patients with SpA showed elevated office BP, higher FBG, and decreased HDL-C concentration, suggesting a diabetic-like pattern of findings, despite frequent treatment with statins, beta-blockers, and ACE inhibitors/ARBs. However, the use of antidiabetic medication did not differ between patients and controls, suggesting that untreated hyperglycemia may promote inflammation and pain intensity in patients with SpA, as previously shown [47,48]. This study was also dominated by women with an average age of around 55 years, who were more frequently diagnosed with knee osteoarthritis, which is usually accompanied by hyperglycemia, dyslipidemia, and arterial hypertension—conditions that pave the way for CV disease [49]. It follows that the same pro-inflammatory mechanisms overlap in metabolic syndrome, CV disease, and SpA.

Here we show that more than one-third of patients with SpA had impaired LV function. Left atrial deformation analysis and tissue Doppler imaging, together with the full set of diastolic parameters, revealed that the E-wave, e′ wave, E/A ratio, LASr, and SrE were significantly lower, while IVRT was higher, in patients with SpA compared with controls. Consistent with this, patients with r-axSpA had a worse E/A ratio and prolonged deceleration time and mean IVRT compared with controls, indicating diastolic dysfunction [22]. We also found decreased parameters of LV systolic function (LVEF, GLS, and MAPSE) in patients with SpA compared with controls, consistent with recent findings by Baniaamam et al. [50]. These findings demonstrate that diastolic function—primarily parameters of early myocardial relaxation—shows a substantially greater degree of subclinical impairment than systolic function [51]. Advanced tissue deformation analysis reveals functional alterations well before major volumetric changes occur in patients with axial SpA [52]. Indeed, LVEDD did not change significantly in our patients. Notably, the extent of this impairment is strictly dependent on age and sex, as advanced age and postmenopausal status in women further exacerbate the decline in diastolic parameters through the synergistic effects of aging and chronic inflammation [53].

Inflammation is a key driver in the development of various heart failure phenotypes [23], including heart failure in patients with SpA [54]. Unresolved inflammation in SpA leads to left atrial and ventricular remodeling through immune-mediated myocarditis [54,55] and, over time, to fibrosis with diastolic and/or systolic heart failure [56]. Inflammatory cardiomyopathy—that is, heart failure without a history of CV disease—is more common in patients with SpA than in healthy controls [51].

In practice, inflammation in patients with SpA is assessed through disease activity parameters based on pain assessment. Axial and peripheral pain are inherent features of SpA that become more pronounced during disease activation and worsen comorbidities and the patient’s overall health [57]. Therefore, the patient’s subjective assessment of pain and the more objective composite indices for assessing predominantly axial activity (BASDAI and ASDAS) [28] and predominantly peripheral activity (DAPSA) [27] essentially reflect inflammation, although pain itself is assessed clinically [57]. ASDAS appeared to be weakly and negatively correlated with MAPSE in univariate analysis. In multivariate analysis, this correlation was independent of vitamin D; however, metabolic factors (glucose and uric acid) significantly influenced the relationship between ASDAS and MAPSE, and the correlation became nonsignificant in the multivariate model. Thus, the observed correlation between ASDAS and MAPSE can be entirely explained by confounding factors in our multivariate model. Accordingly, the positive correlation between HbA1c and parameters of mitral inflow and tissue Doppler echocardiography in patients without diabetes, observed in univariate analysis, disappeared after controlling for age, sex, systolic BP, hypertension, cigarette smoking, BMI, and LDL-C [58]. This loss of significance between ASDAS and MAPSE in multivariate analysis contrasts with the notion that subclinical early diastolic dysfunction is related to inflammation [51,59]. However, ASDAS and DAPSA were significantly associated with prolonged IVRT even after controlling for NSAID and statin therapy, age, sex, and LDL-C or HDL-C, indicating a significant association between inflammation and diastolic dysfunction, as reported by other researchers [22,51,59], although a causal relationship between these parameters cannot be inferred from correlation analyses. Additionally, DAPSA was negatively correlated with the e′ wave. The literature shows marked discrepancy in the reported prevalence of diastolic dysfunction in patients with SpA using the tissue Doppler parameter e′, ranging from 16.6% [22] to 53% [50]. According to our data, the e′ wave emerged as a significant and independent predictor of DAPSA, even after adjusting for lipid parameters (LDL-C and HDL-C) and statin therapy, neither of which showed a significant effect.

Patients with SpA—especially those with PsA, who accounted for 48% of the study group—showed a pronounced metabolic syndrome, which in itself represents a high CV burden in PsA [60], since the degree of diastolic dysfunction may be independently associated with metabolic syndrome [20]. About 20% of patients with PsA have hyperlipoproteinemia, 40% have obesity, 70% are at risk of diabetes, and increased carotid intima-media thickness and asymptomatic heart failure are more frequent [61]. Patients with psoriasis may also have asymptomatic cardiomyopathy even in the absence of traditional CV risk factors [61]. It is now considered that the total CV risk present in 43% of patients with PsA is associated equally with classic CV risk factors and inflammatory conditions [60,61].

In patients with PsA, we found that ASDAS and DAPSA were significantly correlated with one another independently of FBG, LDL-C, and statin and NSAID use, which was not the case when all SpA subtypes were pooled together. Notably, 25–70% of patients with PsA develop the axial form of the disease [62]. However, an increase in ASDAS, but not DAPSA, was associated with worsening systolic LV function, measured by MAPSE and LVEF, in univariate analyses. This association remained significant after controlling for FBG and statin use, confirming more severe disease in patients with axial PsA than in peripheral PsA [62]. The only significant correlation between DAPSA and echocardiographic parameters was with the E-wave, which became nonsignificant after controlling for FBG and statin therapy.

Vitamin D deficiency is associated with the highest risk of CV disease incidence [63], owing to the absence of cardioprotective, anti-inflammatory, and antifibrotic signaling pathways [64] mediated by 25(OH)D3 binding to its widely distributed receptors on cardiac myocytes, endothelial cells, and fibroblasts [65]. Lower vitamin D levels, found in up to 90% of patients with chronic heart failure, are associated with adverse LV remodeling [65], involving structural and functional changes that lead to diastolic and, subsequently, systolic heart failure [64]. Low vitamin D concentrations (below 40–50 nmol/L) have been observed in patients with active r-axSpA [66] and in patients with PsA [67]. In this study, patients with SpA and vitamin D insufficiency showed worse diastolic parameters (LASr, IVRT, and the e′ wave), lower MAPSE and impaired GLS—indicating LV systolic impairment [68]—and a worse lipid profile than patients with sufficient vitamin D. This is consistent with findings that vitamin D supplementation reduces extracellular fibrotic deposition and improves myocardial contraction and relaxation [64,69]. However, the association between vitamin D and better (more negative) GLS observed in univariate analysis disappeared after adjusting for sex, age, uric acid, creatinine, and cigarette smoking. Similarly, the positive correlation between vitamin D and LASr—a reliable and feasible echocardiographic parameter closely correlated with the severity of both diastolic dysfunction and clinical heart failure [51]—disappeared after adjusting for HDL-C, LDL-C, FBG, and statin use. In patients with SpA, the significant positive association between vitamin D and the e′ wave, a key tissue Doppler measure of diastolic function [33,34], was independent of age, sex, LDL-C, HDL-C, and statin use in a multivariate analysis model. However, association does not equal causation, and it remains an open question whether a stronger immune response in the context of vitamin D insufficiency can cause causally worsen cardiac performance in patients with SpA.

We also found no correlation between vitamin D and subjective indices of SpA activity, such as the patient’s perception of SpA activity, total and axial pain, or the composite BASDAI index. Durmus et al. [70] and Cai et al. [71] previously showed that pain and BASDAI were inversely correlated with serum vitamin D in patients with r-axSpA, who represented only 46% of our SpA study group. However, it was recently reported that vitamin D was not associated with pain or disease activity in Chinese patients with axSpA [25], which aligns more closely with our results, given that axSpA patients represented 64% of our study group. We also found no correlation between vitamin D and ESR—a more objective laboratory parameter of SpA activity [70]—or CRP, including composite scales incorporating CRP, such as ASDAS and DAPSA [26,27]. Previously, an inverse correlation was found between vitamin D and both ESR and CRP in patients with r-axSpA, a subgroup of SpA patients [70]. An inverse correlation between vitamin D and DAPSA was demonstrated in patients with pSpA [16], who made up only 36% of our SpA study group, which may explain why DAPSA showed only a trend toward a negative correlation with vitamin D in our patients with PsA. In patients with PsA, the negative correlation between vitamin D and DAPSA became nonsignificant after controlling for FBG, LDL-C, and statin and NSAID use. Finally, we found no association between serum vitamin D and subjective parameters of SpA functionality (BASFI), consistent with Kolahi and colleagues [72].

From the above, it follows that vitamin D did not affect SpA activity or patient functionality, as measured in this real-life study in which more than one-third of patients received vitamin D supplementation.

The main weaknesses of this research are its observational and cross-sectional design, which precludes investigation of causal relationships, and the large number of exploratory analyses, which increases the possibility of type I error. The high CV burden in patients with SpA also complicates interpretation of data related to vitamin D and proinflammatory CV risk factors. Another limitation was the difficulty of recruiting equal or sufficiently large numbers of patients in particular subgroups from a single rheumatology center. The study cohort therefore comprises various SpA subtypes, which may differ in pathophysiology, inflammatory burden, and treatment; pooling all subtypes together may obscure subtype-specific associations and hinder comparison of our data with those of other studies, although the phenotype of our SpA patients is described in detail, and the significance threshold for correlation coefficients was set at r > 0.25. We also present here an analysis of correlations among vitamin D, ASDAS, and DAPSA with echocardiographic parameters in patients with PsA specifically, which should be expanded with more subjects. This research was conducted at a single center, underscoring the need to extend it to multiple centers to help remove possible bias in clinical, laboratory, and radiological examinations.

## 5. Conclusions

In this study, we observed that patients with SpA exhibited significant LV impairment compared with controls. This was evidenced by impaired diastolic function—characterized by elevated IVRT alongside reduced E-wave velocity, e′ wave, and LASr—as well as decreased systolic performance, indicated by lower MAPSE, GLS, and LVEF.

The inflammatory parameters ASDAS and DAPSA were associated with prolongation of IVRT after adjustment for age, sex, LDL-C, NSAID, and/or statin use, and DAPSA was inversely correlated with the e′ wave after controlling for LDL-C, HDL-C, and statin use in patients with SpA. The inverse correlation between ASDAS and MAPSE disappeared after adjustment for metabolic factors, systolic BP, and statin use. Only the e′ wave was positively correlated with vitamin D, and this correlation was independent of age, sex, metabolic factors (LDL-C, HDL-C), NSAID, and statin therapy in patients with SpA. The weak correlations between the echocardiographic parameters GLS and LASr and vitamin D disappeared after adjusting for metabolic factors, age, sex, and statin and NSAID therapy. Serum vitamin D concentration was not associated with ASDAS, DAPSA, or BASFI. Patients with SpA and vitamin D insufficiency demonstrated greater impairment of LV diastolic function (LASr, IVRT, and e′ velocity) and reduced GLS compared with those with sufficient vitamin D.

It follows from the above that vitamin D was not associated with SpA activity. Patients with insufficient serum vitamin D levels (<75 nmol/L) showed greater impairment of systolic and diastolic LV function and a diabetic-like lipid profile compared with those with sufficient vitamin D. SpA activity, expressed through ASDAS and DAPSA, was positively correlated with IVRT, and DAPSA was additionally inversely correlated with the e′ wave, after adjustment for age, sex, metabolic factors, and/or therapy. The e′ wave was positively correlated with vitamin D when confounding factors were controlled.

These main findings in patients with SpA appear consistent with findings in the subgroup of patients with PsA, as follows: (I) ASDAS and DAPSA were significantly correlated with one another; (II) ASDAS was inversely correlated with LVEF and MAPSE after controlling for FBG and/or statin therapy; (III) vitamin D was positively correlated with e′ and negatively correlated with E/e′; and (IV) vitamin D was not independently associated with ASDAS in patients with PsA. However, causal conclusions cannot be drawn from correlational analyses.

## Figures and Tables

**Table 1:** Clinical, laboratory and echocardiographic characteristics of patients with SpA and controls.

	Patients with SpA (n = 298)	Controls (n = 57)	Student’s *t* Test *p* Value
	Mean ± SD	Mean ± SD	
Age (years)	56 ± 11.9	53.15 ± 11.69	0.1131
BMI (kg/m^2^)	27.22 ± 4.78	26.33 ± 2	0.6138
ESR (mm/h)	11.48 ± 8.69	13.59 ± 12.65	0.1314
CRP (mg/L)	2.79 ± 3.31	2.04 ± 2.03	0.3775
Total cholesterol (mmol/L)	5.51 ± 1.14	5.27 ± 1.06	0.1754
HDL-C (mmol/L)	1.66 ± 0.48	1.75 ± 0.64	0.0003 *
FBG (mmol/L)	5.56 ± 0.85	5.15 ± 0.62	0.0014 *
Uric acid (μmol/L)	231.5 ± 101	258.7 ± 73.66	0.0682
eGFR (mL/min/1.73 m^2^)	87.12 ± 13.69	89.44 ± 16.42	0.2973
Vitamin D (nmol/L)	64.4 ± 21.82	62.47 ± 26.14	0.6360
Systolic BP (mmHg)	129.1± 19.6	123.72 ± 14.92	0.0060 *
Diastolic BP (mmHg)	81.24 ± 11.5	78.1 ± 9.67	0.0155 *
SCORE 2/SCORE 2OP	4.66 ± 3.9	6.57 ± 5.88	0.1344
LVEF (%)	58 ± 4.9	60.22 ± 6.24	0.0083 *
MAPSE (mm)	12.59 ± 1.74	13.66 ± 2.39	0.0024 *
GLS (%)	−19.6 ± 2.83	−21.25 ± 2.2	0.0037 *
LVEDD (mm)	50.17 ± 8.14	48.17 ± 3.8	0.0768
Wave A (m/s)	0.78 ± 0.17	0.72 ± 0.17	0.6147
Wave E (m/s)	0.66 ± 0.14	0.81 ± 0.17	<0.0001 *
Ratio E/A	0.96 ± 0.31	1.12 ± 0.49	0.0037 *
LAVI ml/m^2^	15.45 ± 3.21	16.31 ± 1.6	0.0687
	Median (range)	Median (range)	Man-Whitney U Test *p* Value
LASr (%) #	27 (9.56–56)	40 (14–59)	<0.0001 *
Wave e′ (m/s) #	0.08 (0.04–0.17)	0.13 (0.06–0.19)	0.0364 *
Ratio E/e′ #	8.22 (3.46–18)	8 (3.93–14)	0.6528
SrE (1/s) #	−1 [−2.46–(1)]	−2.1 [−3.59–(−0.8)]	<0.0001 *
SrA (1/s) #	−1.46 (−2.05–0.5)	−1.43 [−3.38–(−0.64)]	1.0000
IVRT ms #	121 (64–173)	78 (60–91)	<0.0001 *
Troponin T (ng/L) #	5.24 (3–30.3)	5.87 (3–23)	0.7268
NT-proBNP (pmol/L) #	60 (5–595)	65.5 (13–400)	0.9166
Klotho #	3.08 (0.56–31.1)	3.73 (2.1–5.87)	0.3974

Abbreviations: BMI—body mass index; CRP—C-reactive protein; eGFR—estimated glomerular filtration rate; ESR—erythrocyte sedimentation rate; FBG—fasting blood glucose; GLS—global systolic longitudinal strain; HDL-C—high-density lipoprotein cholesterol; IVRT—isovolumic relaxation time; LASr—peak systolic left atrial reservoir strain; LAVI—left atrial volume indexed to the body surface area; LVEDD—left ventricle end diastolic diameter; LVEF—left ventricle ejection fraction; MAPSE—Mitral Annular Plane Systolic Excursion; n—number; NT-proBNP—N-terminal pro-Brain Natriuretic peptide; %—percentage; SD—standard deviation; SpA—spondyloarthritis; SrA—peak strain rate of left atrium contractile phase; SrE—peak strain rate of the left atrium conduit phase; Wave A—late peak diastolic mitral inflow velocity; wave E—early peak diastolic mitral inflow velocity; wave e—early diastolic mitral annular velocity; SCORE 2/SCORE 2OP –systematic coronary risk evaluation, age 40–69 years/older persons, age 70–89 years. * Statistical significance (*p*) ≤ 0.05 considered significant in all tests performed. The number of subjects for individual parameters in the group of patients with SpA ranged from 58 to 298, and in the control group it was from 21 to 57. The sign # indicates groups with n < 50, which were analyzed by the Mann–Whitney U test, and the other groups by Student’s *t* test.

**Table 2:** Comorbidities and current therapy of the patients with SpA and controls.

Comorbidities and Current Therapy	Patients with SpA, n (%)	Controls n (%)	Fisher’s Exact Test *p* Value
Arterial hypertension	111 (37.24)	0 (0)	<0.0001 *
Hyperglycemia	26 (8.72)	1 (1.79)	0.0483 *
Osteoarthritis	78 (26.17)	1 (1.75)	<0.0001 *
Cigarette smoking	50 (16.77)	1 (1.75)	0.0008 *
Vitamin D supplementation	112 (37.58)	2 (3.5)	<0.0001 *
Hiperlipidemia	128 (42.95)	21 (36.8)	0.2398
Statin	57 (19.12)	4 (7.01)	0.0156 *
NSAID	186 (62.61)	4 (7.01)	<0.0001 *
csDMARD	134 (44.96)	0 (0)	<0.0001 *
bDMARD	23 (7.71)	0 (0)	0.0155 *
Beta blockers	64 (21.47)	4 (7.01)	0.0057 *
ACE inhibitors/ARBs	75 (25.16)	8 (14.03)	0.0452 *
Calcium antagonist	28 (9.39)	5 (8.77)	0.5582
Diuretics	25 (8.38)	3 (5.26)	0.3108
Antidiabetics	9 (3.02)	0 (0)	0.1938

Abbreviations: ACE—Angiotensin-Converting Enzyme; ARBs—Angiotensin II Receptor Blockers; bDMARD—biologic Disease-Modifying Antirheumatic Drug; csDMARD—conventional synthetic Disease-Modifying AntiRheumatic Drug; NSAID—Nonsteroidal Anti-Inflammatory Drug; n—number; SpA—Spondyloarthritis. * Statistical significance (*p*) ≤ 0.05 considered significant in all tests performed.

**Table 3:** Comparison of echocardiographic and metabolic parameters in patients with SpA classified into groups with respect to the cut-off vitamin D concentration (75 nmol/L).

	Vitamin D <75 nmol/L (n = 183)	Vitamin D >75 nmol/L (n = 77)	Student’s *t* Test
	Mean ± SD	Mean ± SD	*p* Value
Tryglicerides (mmil/L)	1.43 ± 0.64	1.21 ± 0.47	0.010 *
HDL-C(mmil/L)	1.45 ± 0.4	1.64 ± 0.38	0.0009 *
LDL-C (mmol/L)	3.56 ± 0.96	3.29 ± 1.07	0.0548
Total cholesterol (mmil/L)	5.56 ± 1.1	5.36 ± 1.29	0.2259
FBG (mmil/L)	5.57 ± 0.85	5.44 ± 0.66	0.3122
Uric acid (μmol/L)	221.54 ± 104.05	232.6 ± 93.91	0.440
Creatinine (μmol/L)	72.76 ± 17.43	72.69 ± 13.47	0.9766
LVEF (%)	57.87 ± 5.06	58.98 ± 382	0.1888
LVEDD (mm)	50.06 ± 8.49	49.31 ± 4.74	0.5776
MAPSE (mm)	12.29 ± 1.65	13.1 ± 1.6	0.0166 *
GLS (%)	−19.22 ± 3.06	−20.45 ± 2	0.0421 *
Wave E (m/s)	0.65 ± 0.14	0.65 ± 0.12	0.9159
Wave A (m/s)	1.69 ± 0.15	0.72 ± 0.19	0.4210
E/A ratio	0.98 ± 0.26	0.94 ± 0.36	0.4873
			Man-Whitney U test
	Median (range)	Median (range)	*p* value
TR velocity (m/s) #	2.2 (1.19–2.97)	2 (0.67–2.96)	0.6027
LAVI (mL/m^2^) #	15.7 (10–22.1)	13.6 (11.3–24.8)	0.4260
SrE (1/s) #	−0.94 (−2.46–(−0.45)	−1 [(−2.16)–(−0.1)]	0.9539
SrA (1/s) #	−1.51 (−2.04–0.52)	−1.35 [(−2.05)–(−0.86)]	0.4882
E/e′ ratio #	8.57 (4.52–16.85)	8 (3.46–12.8)	0.1521
Wave e′ (m/s) #	0.08 (0.04–0.14)	0.09 (0.07–0.17)	0.0044 *
LASr (%) #	26.25 (9.56–41)	32 (18–56)	0.0050 *
IVRT (ms) #	115.5 (64–169)	129. (78–173)	0.0052 *

Abbrevations: FBG—fasting blood glucose; GLS—global systolic longitudinal strain; HDL-C—high-density lipoprotein-cholesterol; IVRT—isovolumic relaxation time; LASr—peak systolic left atrial reservoir strain; LAVI—left atrial volume indexed to the body surface area; LDL-C—Low density lipoprotein—cholesterol; LVEDD—left ventricle end diastolic diameter; LVEF—left ventricle ejection fraction; MAPSE—Mitral Annular Plane Systolic Excursion; n—number; %—percentage; SD—standard deviation; SpA—spondyloarthritis; SrA—peak strain rate of left atrium contractile phase; SrE—peak strain rate of the left atrium conduit phase; TR velocity—maximal velocity of tricuspid regurgitation; Wave A—late peak diastolic mitral inflow velocity; wave E—early peak diastolic mitral inflow velocity; wave e—early diastolic mitral annular velocity. The number of subjects for individual parameters in the group of patients with vitamin D values < 75 nmol/L was from 32 to 179, and in the group of patients with vitamin D values > 75 nmol/L it was from 19 to 73. The sign # indicates groups with n < 50, which were analyzed by the Mann–Whitney U test, while the other samples were analyzed by Student’s *t* test. * Statistical significance at (*p*) ≤ 0.05 was considered significant.

## Data Availability

Data are available upon request.

## Supplementary Materials

The following supporting information can be downloaded at: https://www.mdpi.com/article/10.3390/jcm15156017/s1, Table S1: Correlation of cardiac parameters and parameters of spondiloarthritis [SpA] activity and vitamin D, Axial Spondyloarthritis Disease Activity Score [ASDAS] and Disease Activity Index for Psoriatic Arthritis [DAPSA] in patients with SpA; Table S2: Correlation of cardiac parameters and parameters of spondiloarthritis [SpA] activity and vitamin D, Axial Spondyloarthritis Disease Activity Score [ASDAS] and Disease Activity Index for Psoriatic Arthritis [DAPSA] in subgroup of patients with psoriatic arthritis.

## Abbreviations

The following abbreviations are used in this manuscript:

25OHD3	25-hydroksicholecalciferol
ACE	angiotensin-converting enzyme
ARB	angiotensin II receptor blocker
ASDAS	Ankylosing Spondylitis Disease Activity Score
axSpA	Axial spondyloarthritis
BASDAI	Bath Ankylosing Spondylitis Disease Activity Index
BASFI	Bath Ankylosing Spondylitis Functional Ind
bDMARD	biologic disease-modifying antirheumatic drug
BMI	body mass index
CRP	C-reactive protein
csDMARD	conventional synthetic disease-modifying antirheumatic drug
CV	cardiovascular
DAPSA	Disease Activity Index for Psoriatic Arthritis
E	wave early peak diastolic mitral inflow velocity
e′	early diastolic mitral annular velocity
eGFR	estimated glomerular filtration rate
ELISA	enzyme-linked immunosorbent assay
ESR	erythrocyte sedimentation rate
FBG	fasting blood glucose
GLS	global systolic longitudinal strain
HDL C	high-density lipoprotein cholesterol
IVRT	isovolumic relaxation time
LASr	peak systolic left atrial reservoir strain
LAVI	left atrial volume indexed to the body surface area
LDL-C	low-density lipoprotein cholesterol
LV	left ventricle
LVEDD	left ventricle end diastolic diameter
LVEF	left ventricle ejection fraction
MAPSE	Mitral Annular Plane Systolic Excursion
NRS	numeric rating scale
NSAR	nonsteroidal anti-inflammatory drugs
NT- proBNP	N-terminal pro B-type natriuretic peptide
pSpA	peripheral spondyloarthritis
SCORE 2/SCORE 2OP	systematic coronary risk evaluation
SpA	spondyloarthritis
SrA	peak strain rate of left atrium contractile phase
SrE	peak strain rate of the left atrium conduit phase
TR	tricuspid regurgitation
VAS	visual analog scale

## References

[1] Hebeisen M., Micheroli R., Scherer A., Baraliakos X., de Hooge M., van der Heijde D., Landewé R., Bürki K., Nissen M.J., Möller B. (2020). Spinal radiographic progression in axial spondyloarthritis and the impact of classification as onradiographic versus radiographic disease: Data from the Swiss Clinical Quality Management cohort. PLoS ONE.

[2] Van Gaalen F.A., Rudwaleit M. (2023). Challenges in the diagnosis of axial spondyloarthritis. Best Pract. Res. Clin. Rheumatol..

[3] Puche-Larrubia M.Á., López-Medina C., Ziadé N. (2023). Peripheral spondyloarthritis: What have we learned?. Best Pract. Res. Clin. Rheumatol..

[4] Ziade N., Rassi J., Elzorkany B., Lopez-Medina C., Gamal S.M., Hlais S., Dougados M., Baraliakos X. (2022). What is peripheral spondyloarthritis? Identifying proportion, phenotype and burden in post hoc analysis of the ASAS-PerSpA study. Semin. Arthritis. Rheum..

[5] Shahid Z., Brent L.H., Lucke M. (2024). Enteropathic Arthritis. StatPearls.

[6] Mauro D., Cai B., Ciancio A., Forte G., Gandolfo S., Thomas R., Bergot A.S., Ciccia F. (2025). The role of the gut and intestinal dysbiosis in the pathogenesis of spondyloarthritis. Jt. Bone Spine.

[7] Athanassiou L., Kostoglou-Athanassiou I., Koutsilieris M., Shoenfeld Y. (2023). Vitamin D and autoimmune rheumatic diseases. Biomolecules.

[8] de la Guía-Galipienso F., Martínez-Ferran M., Vallecillo N., Lavie C.J., Sanchis-Gomar F., Pareja-Galeano H. (2021). Vitamin D and cardiovascular health. Clin. Nutr..

[9] Alonso N., Zelzer S., Eibinger G., Herrmann M. (2023). Vitamin D metabolites: Analytical challenges and clinical relevance. Calcif. Tissue Int..

[10] Ao T., Kikuta J., Ishii M. (2021). The effects of vitamin D on immune system and inflammatory diseases. Biomolecules.

[11] Xu Y., Sun Z. (2015). Molecular basis of Klotho: From gene to function in aging. Endocr. Rev..

[12] Veldman C.M., Cantorna M.T., DeLuca H.F. (2000). Expression of 1,25-dihydroxyvitamin D3 receptor in the immune system. Arch. Biochem. Biophys..

[13] Artusa P., White J.H. (2025). Vitamin D and its analogs in immune system regulation. Pharmacol. Rev..

[14] Tsukazaki H., Kaito T. (2020). The role of the IL-23/IL-17 pathway in the pathogenesis of spondyloarthritis. Int. J. Mol. Sci..

[15] Bosch P., Zhao S.S., Nikiphorou E. (2023). The association between comorbidities and disease activity in spondyloarthritis: A narrative review. Best Pract. Res. Clin. Rheumatol..

[16] Castro Corredor D., Ramírez Huaranga M.A., Mínguez Sánchez M.D., Anino Fernández J., Mateos Rodríguez J.J., Rebollo Giménez A.I., González Peñas M., Seoane Romero J., Luque Zafra M., de Lara Simón I.M. (2020). Vitamin D, an inflammatory activity marker for spondyloarthritis?. Arch. Osteoporos..

[17] Ben-Shabat N., Watad A., Shabat A., Bragazzi N.L., Comaneshter D., Cohen A.D., Amital H. (2020). Low vitamin D levels predict mortality in ankylosing spondylitis patients: A nationwide population-based cohort study. Nutrients.

[18] Issakulova A., Yessirkepov M., Zimba O., Kocyigit B.F. (2025). Cardiovascular risk in axial spondyloarthritis: The interplay of inflammation, traditional risk factors, and management strategies. Rheumatol. Int..

[19] Garshick M.S., Ward N.L., Krueger J.G., Berger J.S. (2021). Cardiovascular risk in patients with psoriasis: JACC review topic of the week. J. Am. Coll. Cardiol..

[20] Cioffi G., Viapiana O., Tarantini L., Orsolini G., Idolazzi L., Sonographer F.O., Dalbeni A., Gatti D., Fassio A., Rossini M. (2021). Clinical profile and outcome of patients with chronic inflammatory arthritis and metabolic syndrome. Intern. Emerg. Med..

[21] Ntusi N.A., Piechnik S.K., Francis J.M., Ferreira V.M., Rai A.B., Matthews P.M., Robson M.D., Moon J., Wordsworth P.B., Neubauer S. (2014). Subclinical myocardial inflammation and diffuse fibrosis are common in systemic sclerosis—A clinical study using myocardial T1-mapping and extracellular volume quantification. J. Cardiovasc. Magn. Reson..

[22] Bolaji O., Oriaifo O., Adabale O., Dilibe A., Kuruvada K., Ouedraogo F., Ezeh E., Nair A., Olanipekun T., Mazimba S. (2024). A meta-analysis of left ventricular dysfunction in ankylosing spondylitis. J. Clin. Hypertens..

[23] Toussirot E. (2021). The risk of cardiovascular diseases in axial spondyloarthritis: Current insights. Front. Med..

[24] Jiang J., Shao M., Wu X. (2022). Vitamin D and risk of ankylosing spondylitis: A two-sample Mendelian randomization study. Hum. Immunol..

[25] Deng S., He Y., Nian X., Sun E., Li L. (2019). Relationship between vitamin D levels and pain and disease activity in patients with newly diagnosed axial spondyloarthritis. Int. J. Nurs. Sci..

[26] Ørnbjerg L.M., Georgiadis S., Kvien T.K., Michelsen B., Rasmussen S., Pavelka K., Zavada J., Loft A.G., Kenar G., Solmaz D. (2024). Impact of patient characteristics on ASDAS disease activity state cut-offs in axial spondyloarthritis: Results from nine European rheumatology registries. RMD Open.

[27] Smolen J.S., Schoels M., Aletaha D. (2015). Disease activity and response assessment in psoriatic arthritis using DAPSA. A brief review. Clin. Exp. Rheumatol..

[28] Rudwaleit M., van der Heijde D., Landewé R., Akkoc N., Brandt J., Chou C.T., Dougados M., Huang F., Gu J., Kirazli Y. (2011). The Assessment of SpondyloArthritis International Society classification criteria for peripheral spondyloarthritis and for spondyloarthritis in general. Ann. Rheum. Dis..

[29] World Medical Association (2025). World Medical Association Declaration of Helsinki: Ethical Principles for Medical Research Involving Human Participants. JAMA.

[30] Jones D.W., Ferdinand K.C., Taler S.J., Johnson H.M., Shimbo D., Abdalla M., Altieri M.M., Bansal N., Bello N.A., Bress A.P. (2025). 2025 AHA/ACC/AANP/AAPA/ABC/ACCP/ACPM/AGS/AMA/ASPC/NMA/PCNA/SGIM Guideline for the Prevention, Detection, Evaluation and Management of High Blood Pressure in Adults: A Report of the American College of Cardiology/American Heart Association Joint Committee on Clinical Practice Guidelines. Hypertension.

[31] McDonagh T.A., Metra M., Adamo M., Gardner R.S., Baumbach A., Böhm M., Burri H., Butler J., Čelutkienė J., Chioncel O. (2022). 2021 ESC Guidelines for the diagnosis and treatment of acute and chronic heart failure: Developed by the Task Force for the diagnosis and treatment of acute and chronic heart failure of the European Society of Cardiology (ESC). With the special contribution of the Heart Failure Association (HFA) of the ESC. Eur. J. Heart Fail..

[32] Michielsens C., Bolhuis T.E., van Gaalen F.A., van den Hoogen F., Verhoef L.M., den Broeder N., den Broeder A.A. (2024). Construct validity of BASDAI and ASDAS treatment target cut-offs in axial spondy-loarthritis. Scand. J. Rheumatol..

[33] Lang R.M., Badano L.P., Mor-Avi V., Afilalo J., Armstrong A., Ernande L., Flachskampf F.A., Foster E., Goldstein S.A., Kuznetsova T. (2015). Recommendations for cardiac chamber quantification by echocardiography in adults: An update from the American Society of Echocardiography and the European Association of Cardiovascular Imaging. Eur. Heart J. Cardiovasc. Imaging.

[34] Nagueh S.F., Smiseth O.A., Appleton C.P., Byrd B.F., Dokainish H., Edvardsen T., Flachskampf F.A., Gillebert T.C., Klein A.L., Lancellotti P. (2016). Recommendations for the evaluation of left ventricular diastolic function by echocardiography: An update from the American Society of Echocardiography and the European Association of Cardiovascular Imaging. J. Am. Soc. Echocardiogr..

[35] Akoglu H. (2018). User’s guide to correlation coefficients. Turk. J. Emerg. Med..

[36] Ermann J. (2025). Rethinking spondyloarthritis: Beyond lumping and splitting. Curr. Opin. Rheumatol..

[37] Chang M.H., Fuhlbrigge R.C., Nigrovic P.A. (2024). Joint-specific memory, resident memory T cells and the rolling window of opportunity in arthritis. Nat. Rev. Rheumatol..

[38] Mortier C., Quintelier K., De Craemer A.S., Renson T., Deroo L., Dumas E., Verheugen E., Coudenys J., Decruy T., Lukasik Z. (2023). Gut inflammation in axial spondyloarthritis patients is characterized by a marked type 17 skewed mucosal innate-like T cell signature. Arthritis Rheumatol..

[39] Romero-Sanchez C., Jaimes D.A., Londoño J., De Avila J., Castellanos J.E., Bello J.M., Bautista W., Valle-Oñate R. (2011). Association between Th-17 cytokine profile and clinical features in patients with spondyloarthritis. Clin. Exp. Rheumatol..

[40] Knežević D., Ćurko-Cofek B., Batinac T., Laškarin G., Rakić M., Šoštarič M., Zdravković M., Šustić A., Sotošek V., Batičić L. (2023). Endothelial dysfunction in patients undergoing cardiac surgery: A narrative review and clinical implications. J. Cardiovasc. Dev. Dis..

[41] Takeda Y., Matoba K., Sekiguchi K., Nagai Y., Yokota T., Utsunomiya K., Nishimura R. (2020). Endothelial dysfunction in diabetes. Biomedicines.

[42] Batinac T., Batičić L., Kršek A., Knežević D., Marcucci E., Sotošek V., Ćurko-Cofek B. (2024). Endothelial dysfunction and cardiovascular disease: Hyperbaric oxygen therapy as an emerging therapeutic modality?. J. Cardiovasc. Dev. Dis..

[43] Issakulova A., Mukhamediyarov M. (2026). Clinical presentation and rehabilitation approaches in patients with ankylosing spondylitis and comorbidities: A multicenter retrospective study. Rheumatol. Int..

[44] El Hasbani G., Nassar J.E., Elsayed Ali A.M., Uthman I., Jawad A. (2024). The impact of nicotine smoking on spondyloarthritis and rheumatoid arthritis. Reumatismo.

[45] Dubsky M., Veleba J., Sojakova D., Marhefkova N., Fejfarova V., Jude E.B. (2023). Endothelial dysfunction in diabetes mellitus: New insights. Int. J. Mol. Sci..

[46] Sobchak C., Eder L. (2017). Cardiometabolic disorders in psoriatic disease. Curr. Rheumatol. Rep..

[47] Zhu S., Qian Y. (2012). IL-17/IL-17 receptor system in autoimmune disease: Mechanisms and therapeutic potential. Clin. Sci..

[48] Laškarin G., Peršić V., Kukić S.R., Massari D., Legović A., Boban M., Miškulin R., Rogoznica M., Kehler T. (2016). Can pain intensity in osteoarthritis joint be indicator of the impairment of endothelial function?. Med. Hypotheses.

[49] Zavidić T., Babarović E., Drvar V., Ćurko-Cofek B., Laškarin G. (2025). Pulse wave velocity and knee osteoarthritis severity. Biomedicines.

[50] Baniaamam M., Paulus W.J., Blanken A.B., Nurmohamed M.T. (2018). The effect of biological DMARDs on the risk of congestive heart failure in rheumatoid arthritis: A systematic review. Expert. Opin. Biol. Ther..

[51] Romand X., Adeline F., Dalecky M., Pflimlin A., Bellier A., Barone-Rochette G., Wendling D., Gaudin P., Claudepierre P., Dougados M. (2022). Systematic assessment of heart valves and cardiac function by echocardiography in axial spondyloarthritis: A systematic review and meta-analysis. Jt. Bone Spine.

[52] Chen Y., Chung H.-Y., Zhao C.-T., Wong A., Zhen Z., Tsang H.H., Lau C.S., Tse H.F., Yiu K.H. (2015). Left ventricular myocardial dysfunction and premature atherosclerosis in patients with axial spondyloarthritis. Rheumatology.

[53] Coppi F., Pagnoni G., Grossule F., Nassar A., Maini A., Masaracchia G., Sbarra F., Battigaglia E., Maggio E., Aschieri D. (2025). Gender-Specific Differences in Diastolic Dysfunction and HFpEF: Pathophysiology, Diagnosis, and Therapeutic Strategies. J. Cardiovasc. Dev. Dis..

[54] Adamo L., Rocha-Resende C., Prabhu S.D., Mann D.L. (2020). Reappraising the role of inflammation in heart failure. Nat. Rev. Cardiol..

[55] Rana A., Mahajan V.K., Mehta K.S., Chauhan P.S., Kumar M., Sharma A., Sharma R., Dhattarwal N., Sondhi M. (2021). Cardiomyopathy and echocardiographic abnormalities in Indian patients with psoriasis: Results of a pilot study. Int. J. Clin. Pract..

[56] Agca R., Smulders Y., Nurmohamed M. (2022). Cardiovascular disease risk in immune-mediated inflammatory diseases. Heart.

[57] Drouet J., López-Medina C., Granger B., Fautrel B., Landewé R.B.M., Molto A., Gaujoux-Viala C., Kiltz U., Dougados M., Gossec L. (2024). Disease activity and widespread pain are main contributors to patient-reported global health in axial spondyloarthritis: An analysis of 6064 patients. Rheumatol. Int..

[58] Jakubiak G.K., Pawlas N., Starzak M., Blachut D., Chwalba A., Wojciechowska C., Cieślar G. (2025). Analysis of the relationship between glycated hemoglobin and echocardiographic parameters in patients without diabetes: A retrospective study. J. Clin. Med..

[59] Prasad S., Rivera A., Nishtala A., Pawlowski A.E., Sinha A., Bundy J.D., Chadha S.A., Ahmad F.S., Khan S.S., Achenbach C. (2020). Chronic inflammatory diseases and incident heart failure. J. Am. Coll. Cardiol. HF.

[60] Totolici S., Vrabie A.M., Buzea C.A., Badila E. (2026). Cardiovascular risk in psoriatic arthritis: Mechanisms, risk assessment, and long-term management implications. Int. J. Mol. Sci..

[61] Bursać I.U., Kehler T., Drvar V., Babarović E., Pejčinović V.P., Baršić A.R., Peršić V., Laškarin G. (2022). Predictive value of monocyte chemoattractant protein-1 in the development of diastolic dysfunction in patients with psoriatic arthritis. Dis. Markers.

[62] Gottlieb A.B., Merola J.F. (2021). Axial psoriatic arthritis: An update for dermatologists. J. Am. Acad. Dermatol..

[63] Grant W.B., Boucher B.J., Cheng R.Z., Pludowski P., Wimalawansa S.J. (2025). Vitamin D and cardiovascular health: A narrative review of risk reduction evidence. Nutrients.

[64] Niccoli G., Del Buono M.G. (2019). Vitamin D and left ventricular adverse remodeling: Does association imply causation?. Int. J. Cardiol..

[65] Chen X., Zhao W., Zhao Y., Ma J., Bu H., Zhao Y. (2023). Vitamin D on cardiac function in heart failure: A systematic review and meta-analysis of RCTs. Rev. Cardiovasc. Med..

[66] Chen M., Li W., Li L., Chai Y., Yang Y., Pu X. (2022). Ankylosing spondylitis disease activity and serum vitamin D levels: A systematic review and meta-analysis. Medicine.

[67] Radić M., Đogaš H., Kolak E., Gelemanović A., Nenadić D.B., Vučković M., Radić J. (2023). Vitamin D in psoriatic arthritis—A systematic review and meta-analysis. Semin. Arthritis Rheum..

[68] Aghdashi M.A., Mikaeilvand A., Ansari-Ramandi M.M., Khodaparast A., Rostamzadeh A. (2023). Speckle tracking echocardiography in ankylosing spondylitis. Mediterr. J. Rheumatol..

[69] Witte K.K., Byrom R., Gierula J., Paton M.F., Jamil H.A., Lowry J.E., Gillott R.G., Barnes S.A., Chumun H., Kearney L.C. (2016). Effects of vitamin D on cardiac function in patients with chronic heart failure: The VINDICATE study. J. Am. Coll. Cardiol..

[70] Durmus B., Altay Z., Baysal O., Ersoy Y. (2012). Vitamin D and disease severity in ankylosing spondylitis. Chin. Med. J..

[71] Cai G., Wang L., Fan D., Xin L., Liu L., Hu Y., Ding N., Xu S., Xia G., Jin X. (2015). Vitamin D in ankylosing spondylitis: Review and meta-analysis. Clin. Chim. Acta.

[72] Kolahi S., Khabbazi A., Kazemi N., Malek Mahdavi A. (2019). Vitamin D deficiency and disease activity in spondyloarthritis. Immunol. Lett..

