# Supplementary material for: Relationship of Vitamin D, Spondyloarthritis Activity and Left Ventricular Systolic and Diastolic Function
Source: J Clin Med. 2026 Aug 2;15(15):6017. doi: 10.3390/jcm15156017 (PMC13467431; doi:10.3390/jcm15156017)
Supplement: Supplementary file 1 [file jcm-15-06017-s001.zip › jcm-4415794-supplementary.pdf]

Supplementary Table S1. Correlation of cardiac parameters and parameters of spondiloarthritis [SpA] activity and vitamin D, Axial Spondyloarthritis Disease Activity Score [ASDAS] and Disease Activity Index for Psoriatic Arthritis [DAPSA] in patients with SpA.

Correlations		P value	r
**Vitamin D (nmol/L)**	ESR (nm)	0.100	-0.102
	CRP (mg/L)	0.041	-0.126
	VAS for total pain (0 – 100 mm)	0.008	-0.163
	VAS axial pain (0 – 10)	0.007	-0.173
	ASDAS	0.014	-0.153
	DAPSA	0.006	-0.248
	BASDAI	0.008	-0.165
	BASFI	0.039	-0.128
	LVEF (%)	0.036	0.171
	LVEDD (mm)	0.087	-0.140
	MAPSE (mm)	0.135	0.146
	IVRT (ms)	0.380	0.115
	E/e′ ratio	0.154	-0.158
**ASDAS**	LVEF (%)	0.028	-0.177
	GLS (%)	0.038	0.205
	LVEDD (mm)	0.013	0.200
	LASr (%)	0.049	-0.225
	E/e′ ratio	0.336	0.109
	Wave e′ (m/s)	0.004	-0.229
**DAPSA**	LVEF (%)	0.197	-0.125
	GLS (%)	0.096	0.192
	LVEDD (mm)	0.334	0.093
	MAPSE (mm)	0.221	-0.134
	LASr (%)	0.064	-0.223
	E/e′ ratio	0.887	-0.018

Abbreviations: BASDAI – Bath Ankylosing Spondylitis Disease Activity Indeks; BASFI – Bath Ankylosing Spondylitis Functional Indeks; CRP – C-reactive protein; e′ – early diastolic mitral annular velocity; ESR – erythrocyte sedimentation rate; GLS – global longitudinal strain; IVRT – isovolumic relaxation time; LVEF – left ventricular ejection fraction; LVEDD – left ventricular end diastolic diameter; MAPSE – mitral annular plane systolic excursion; LASr – peak systolic left atrial reservoir strain; E/e′ – ratio of early transmitral inflow velocity [E] to e′. P [level of statistical significance (Pearson test)]; r [correlation coefficient].

Supplementary Table S2. Correlation of cardiac parameters and parameters of spondiloarthritis [SpA] activity and vitamin D, Axial Spondyloarthritis Disease Activity Score [ASDAS] and Disease Activity Index for Psoriatic Arthritis [DAPSA] in subgroup of patients with psoriatic arthritis

Correlations		P value	r
**Vitamin D (nmol/L)**	LVEF (%)	0.0766	0.215
	MAPSE (mm)	0.1695	0.196
	TR velocity (m/s)	0,5112	0.138
	IVRT (ms)	0.8422	-0.041
	Wave E (m/s)	0.6414	-0.071
	Wave A (m/s)	0.8654	-0.026
	E/A artio	0.6526	0.058
	LAVI (ml/$m^{2)}$	0.2492	-0.240
	LASr (%)	0.0814	0.272
	SrE (s^{-1})	0.9374	0.013
	SrA (s^{-1})	0.2254	-0.191
**ASDAS**	GLS (%)	0.0684	0.272
	TR velocity (m/s)	0.5772	-0.133
	IVRT (ms)	0.1312	0.350
	Wave E (m/s)	0.7324	-0.056
	Wave A (m/s)	0.5563	0.097
	E/A ratio	0.0596	-0.241
	LAVI (ml/m^2)	0.2472	0.264
	LASr (%)	0.1613	-0.239
	Wave e′ (m/s)	0.3583	-0.174
	E/e′ ratio	0.1813	0.251
	SrE (s^{-1})	0.0763	0.296
	SrA (s^{-1})	0.2783	0.183
**DAPSA**	LVEF (%)	0.2437	-0.140
	GLS (%)	0.1804	0.201
	MAPSE (mm)	0.1304	-0.219
	TR velocity (m/s)	0.3292	-0.224
	IVRT (ms)	0.0812	0.399
	Wave A (m/s)	0.6444	-0.075
	Ratio E/A	0.2566	-0.145
	LAVI (ml/m^2)	0.8872	-0.034
	LASr (%)	0.2753	-0.190
	Wave e′ (m/s)	0.0543	-0.355
	E/e′ ratio	0.7393	0.062
	SrE (s^{-1})	0.6733	0.073
	SrA (s^{-1})	0.8113	-0.041

Abbrevations: E/A ratio – ratio of wave A and wave E; E/e′ ratio – ratio of wave E and wave e′; GLS – global longitudinal strain; IVRT – isovolumic relaxation time; LASr – peak systolic left atrial reservoir strain; LAVI – left atrial volume indexed to the body surface area; LVEF – left ventricular ejection fraction; MAPSE – mitral annular plane systolic excursion; SrA – peak strain rate of left atrium contractile phase; SrE – peak strain rate of the left atrium conduit phase; TR velocity – maximal velocity of tricuspid regurgitation; Wave A – late peak diastolic mitral inflow velocity;

Wave E – Early transmitral inflow velocity [E]; Wave e′ – Early diastolic mitral annular velocity [e′]. P [level of statistical significance (Pearson test)]; r [correlation coefficient].
